# Poly(Lactic Acid)–Poly(Butylene Succinate)–Sugar Beet Pulp Composites; Part I: Mechanics of Composites with Fine and Coarse Sugar Beet Pulp Particles

**DOI:** 10.3390/polym13152531

**Published:** 2021-07-30

**Authors:** Rodion Kopitzky

**Affiliations:** Department of Circular and Bio-Based Plastics, Fraunhofer UMSICHT, Fraunhofer Institute for Environmental, Safety and Energy Technology, Osterfelder Str. 3, 46047 Oberhausen, Germany; rodion.kopitzky@umsicht.fraunhofer.de

**Keywords:** sugar beet pulp, polymer composites, bio-based polymers, compatibilization, tensile properties

## Abstract

Sugar beet pulp (SBP) is a residue available in large quantities from the sugar industry, and can serve as a cost-effective bio-based and biodegradable filler for fully bio-based compounds based on bio-based polyesters. The heterogeneous cell structure of sugar beet suggests that the processing of SBP can affect the properties of the composite. An “Ultra-Rotor” type air turbulence mill was used to produce SBP particles of different sizes. These particles were processed in a twin-screw extruder with poly(lactic acid) (PLA) and poly(butylene succinate) (PBS) and fillers to granules for possible marketable formulations. Different screw designs, compatibilizers and the use of glycerol as a thermoplasticization agent for SBP were also tested. The spherical, cubic, or ellipsoidal-like shaped particles of SBP are not suitable for usage as a fiber-like reinforcement. In addition, the fineness of ground SBP affects the mechanical properties because (i) a high proportion of polar surfaces leads to poor compatibility, and (ii) due to the inner structure of the particulate matter, the strength of the composite is limited to the cohesive strength of compressed sugar-cell compartments of the SBP. The compatibilization of the polymer–matrix–particle interface can be achieved by using compatibilizers of different types. Scanning electron microscopy (SEM) fracture patterns show that the compatibilization can lead to both well-bonded particles and cohesive fracture patterns in the matrix. Nevertheless, the mechanical properties are limited by the impact and elongation behavior. Therefore, the applications of SBP-based composites must be well considered.

## 1. Introduction

Sugar beet is one of the most productive crops in the world. According to the data of the Food and Agriculture Organization of the United Nations, the harvested amount added up to approximately 308 million tons in 2019, of which about half came from Europe and one third from the former Russian Federation [1]. The water insoluble residue after the extraction of the sugar, the sugar beet pulp (SBP), consists mainly of approximately equal parts of cellulose, hemicellulose and pectin (in sum 75–85%) and smaller amounts of lignin (<9%), proteins (<7%), lipids (<2%), saponins (<2%) and ash. Approximately 45–50 kg of sugar beet pulp (dry matter) can be obtained from one ton of fresh sugar beet [2,3,4].

The low content of proteins, low-quality primary wall cellulose fibers [2,3,4,5] and the high content of low-quality pectin make further processing into high-quality products difficult. The application as a dietary supplement for animals is therefore the most prominent use [6]. Nevertheless, the valorization of SBP as a solid fuel following a torrefaction process [7], as a substrate for bioethanol production via a fermentation route [8], as a food improver [9,10,11], as a source of micro- and nanofibrils or solubilized cellulose [12,13,14,15], and as a source of pectin [16,17,18], arabinose [19] or ferulic acid [20,21,22] have been investigated.

The valorization of pure SBP as a material was investigated by Rouilly et al. [2,23,24]. By processing SBP in a twin-screw extruder and applying high specific mechanical energy, a thermoplastic-like compound was formed, as indicated by microscopy and by rheological data. The compound is described as a composite made of cellulose micro-fibrils embedded in a matrix constituted of hemicellulose and pectin. Therefore, the breakage of the cell structures is important in the extrusion process. Liu et al. mentioned that the destruction of cell walls is possible without specific design of the screw [25]. They also showed that pectin-extracted SBP can be mixed with pectin (and plasticizer) in different amounts. Thus, pectin can act either as a binder or as a thermoplastic phase. The results were quite similar to those of the native SBP with a similar composition [26]. Nevertheless, the material characterization shows that pure SBP thermoplastics possess an intrinsically high hydrophilicity and high brittleness as well as low moduli and strength values. Due to this poor mechanical behavior and hypersensitivity to water and water vapor, no valuable application has been found yet.

More valuable fully bio-based composite materials made of poly(lactic acid) (PLA) and SBP were investigated by a group of researchers from the National Center for Agricultural Utilization Research from the U.S. Department of Agriculture [27,28,29,30,31,32]. Overall, their results show that unplasticized SBP acts as a filler in PLA but the strengths of the composites are lower than that of pure PLA and the elongation at break and the toughness are not improved. Increasing the proportion of SBP revealed that the mechanics are determined by the size and continuity of the PLA phase rather than by interactions of the interfaces. Acoustic emission analysis of the fracture process showed that the de-bonding of the phases takes place before rupture. The aggregation of SBP particles at high loadings occurs, leading to a lower de-bonding strength [28]. The addition of a coupling agent improves wettability and adhesion and increases mechanical characteristics. Using pre-plasticized SBP results in lower mechanical values, and the de-bonding of the phases occurs at low stresses, as shown by acoustic emission analysis [30]. A high plasticizer content in the pre-plasticized SBP will lead to a co-continuous phase behavior [30]. A fully biodegradable but not fully bio-based composite made of poly(butylene adipate-co-terephthalate) (PBAT) and plasticized SBP is also mentioned by Liu et al. [33]. The SBP stiffens this flexible and soft polyester to some extent at the expense of the good stretchability of pure PBAT.

Shen et al. prepared a composite of acid-treated and bleached SBP with polyvinyl alcohol and a plasticizer for packaging applications [34]. Due to the high cellulose and low pectin content of this treated SBP, the results cannot be compared with composites containing untreated SBP.

The use of fully bio-based and biodegradable composites is advantageous from an ecological point of view when there is a high possibility of plastic parts or fragments thereof remaining in the environment. This applies, for example, to products used in horticulture, agriculture and landscaping. PLA is well known for the fact that its degradation only proceeds quickly above its glass transition temperature [35]. The use of SBP as a filler might increase the biodegradation rate assuming that the nutrient content accelerates the growth of bacteria. Finally, the potential thermoplastic character of SBP can lead to a more homogenous dispersion of SBP in the polymer matrix, which should result in more isotropic properties.

Blends made from PLA and poly(butylene-succinate) (PBS) are often used as a material for injection molding because of easier de-molding and reduced stiffness compared to pure PLA. For instance, compostable coffee capsules are often made of PLA–PBS blends, from nearly pure PLA matrices to nearly pure PBS matrices [36]. In addition, the PBS content may enhance the degradability of PLA at ambient temperature [37].

In this article, we report our work on PLA–PBS–SBP composites focusing on potentially marketable injection molding formulations whose fillers are partially or fully replaced by SBP. In addition to the procedural requirements, the investigations focus on the mechanical properties of SBP composites as a function of the fineness of the SBP particle. The properties of the composites with regard to water absorption will be discussed in Section 2 of this article.

## 2. Materials and Methods

### 2.1. Materials

Polymers: Poly(lactic acid) (Ingeo^TM^ 3251D, NatureWorks LLC, Minnetonka, MN, USA) was purchased from Resinex Germany GmbH (Zwingenberg, Germany). Poly(butylene succinate) (injection molding grade) was a donation from MCPP Europe GmbH (Düsseldorf, Germany). 

Fillers: Chalk was purchased from Omya GmbH (Cologne, Germany). Talc was purchased from Mondo Minerals B.V. (Amsterdam, Netherlands).

Additives: Maleic anhydride modified PLA (PLA-g-MAH, development type) was a donation of BYK-Chemie GmbH (Schkopau, Germany). IPOX^®^ CL 12 (1,2,3-propanetriol-glycidylether) was purchased from IPOX Chemicals GmbH (Laupheim, Germany). Acrylic impact modifier (Biostrength^®^ 150) was purchased from Arkema GmbH (Düsseldorf, Germany). Polyolefin impact modifier (Acti-Tech 16MA13) was a donation of the Nordic Grafting Company A/S (Hellerup, Denmark). All chemicals were used as received.

Ground SBP types were provided by Pfeifer & Langen (Cologne/Elsdorf, Germany) in paper bags. The grinding of the SBP had been accomplished before by Jäckering Mühlen-und Nährmittelwerke GmbH (Hamm, Germany) using an air turbulence mill of type “Ultra-Rotor”. 

We analyzed the water content of the SBP prior to use and noticed that it changed from about 8 to 9 percent after receiving the sample to about 10 to 11 percent before processing depending on humidity conditions. Compound compositions were calculated on a dry mass basis. Due to the natural origin of SBP, deviations in the content of its constituents may occur using SBP from different years and/or different cultivation areas. No analysis of the SBP components was performed.

### 2.2. Compounding

An intermeshing co-rotating twin-screw extruder ZSK 25 from Coperion GmbH (Stuttgart, Germany) with the screw diameter D being 25 mm and the screw length L = 40 D was used for compounding. The polymers were dry blended and fed by a gravimetrical dosing feeder into the hopper. SBP was dry blended with coupling agent and/or fillers and fed by a gravimetrical dosing feeder into a side feeder. The liquid reactive epoxides were uniformly distributed on the SBP, mixed manually and kept at room temperature for at least one hour to allow the reaction between the epoxy groups and the carboxyl groups of the SBP to take place. When using epoxy-modified SBP and the anhydride-modified coupling agent, the coupling agent was added immediately before compounding. 

The extruder consists of eight zones, which can be tempered individually. In the area of the second zone, a liquid dosing connection is installed. A side feeder for the addition of further materials (SBP, filler, coupling agents) follows this. This zone is also equipped with an atmospheric degassing system. At the beginning of the seventh zone, volatile components can be removed from the melt with the aid of a vacuum suction port (~200 mbar). A nozzle plate with two 4 mm diameter nozzle holes completed the process. 

The specific mechanical energy (*SME*) was calculated using Equation (1):

(1) $$SME=6.28\;\frac{t\times r\;}{60\times \dot{m}}$$

with torque *t* (Nm), rotation speed *r* (min^−1^), and throughput $\dot{m}$ (kg hour^−1^). The data collected and stored by the extruder control system every 10 s were averaged over time.

#### 2.2.1. Biopolyester/SBP Composites

Zone temperatures of the extruder were set to 60, 170, 170, 170, 170, 160, 160, and 165 °C for the production of SBP composites with PLA/PBS. The measured temperature of the materials at the die was 176 ± 3 °C (1σ). The strands were water cooled, granulated by an SGS 50-E granulator (Reduction Engineering GmbH, Korntal-Münchingen, Germany) and dried in a dryer (dry air generator, model: LUXOR 80; drying bins: 15 L, Motan-Colortronic GmbH, Kirchlengern, Germany) at 60 °C for several hours until the humidity was below 0.02% (moisture balance MA 30, Sartorius AG, Göttingen, Germany). 

Screw Design 1: In the draw-in zone, push-edge conveyor elements were used to provide a larger free volume. This was to improve the transport of the material and prevent accumulation in the feed hopper. The subsequent conveying elements had a gradient of 1.5 D. This was to facilitate material transport within the screw and reduce the residence time outside the two processing zones to a minimum. Liquids were added in the middle area of the second extruder zone. Here, only conveying elements transported the solids and the liquid. In the area before the first processing zone, extruder zone 4, conveying elements with a gradient of 1.0 D were provided for pre-compressing. The material then passed through a kneading zone with a length of 3.0 D. Three-way kneading elements ensured intensive mixing with homogeneous material loading at the same time. Subsequently, the material was transported to the second processing zone, sector six of the extruder. The task of this zone was the entry of shear into the material. In front of the kneading blocks, conveyor elements with a lower gradient were installed in order to compact the material and build up the necessary pressure for transporting it through the processing zone. First, two kneading elements with a disc offset angle of 45° were used. These elements provided a first distributive and dispersive mixing of the product and, at the same time, further transport through the zone was ensured. Two kneading elements with a neutral offset angle were subsequently applied to significantly increase the shear and the mixing effect. A following return element of 1.0 D length increased the residence time in this zone and prevented the escape of liquids through the vacuum suction before incorporation. The design of the screw was completed by further conveying elements with a large gradient across the vacuum degassing process. Elements with a smaller gradient provided the pressure build-up in front of the nozzle. 

**Figure 1:**
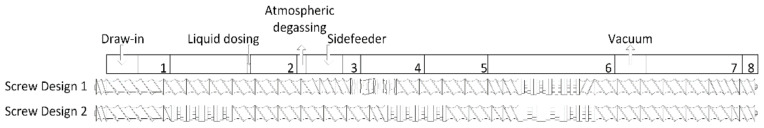
Screw design 1 and 2.

Screw Design 2: To prevent possible degradation of the biopolymers and burning of the SBP by hot spots, a more gentle screw design was applied. Therefore, after the feeding zone, a kneading zone with a smaller disc offset was used. The middle zone also consisted of such elements. Both zones had a length of 4 D. Final mixing before degassing was performed with wider kneading discs and a neutral kneading element of 5 D length. The rest of the screw configuration was comparable to the composite configuration. Schematic representations of both screw designs are shown in Figure 1.

#### 2.2.2. SBP Plasticization

For the plasticization experiments of SBP with water or glycerol, screw design 1 was used. The temperatures of the zones were set to 60, 80, 80, 80, 80, 90, 80 and 55 °C and no die was used. Approx. 1.3 kg SBP (dry mass) per hour was fed to the extruder via the hopper. Based on the SBP dry substance, water was added in the second zone, resulting in a total amount of 45 and 55% water, respectively. Glycerol was added in a total amount of 32.3 and 45%, respectively, based on the dry substance of the SBP. Screw speeds in the range of 150 to 250 rpm maximum were selected in order not to let the torque increase significantly above 50% of the maximum permissible torque.

### 2.3. Measurement of Mechanical Properties

Test specimens for tensile tests were injection molded on a Battenfeld 600 injection molding machine (Wittmann Battenfeld Deutschland GmbH, Meinerzhagen, Germany) equipped with a standard injection molding tool according to ISO 20753:2017 Type 1A (ISO 527 dog-bone-shaped, 2 nests). Die temperature: 170 °C; shot volume: 36–37 cm^3^; injection speed: PLA rich blends: 10 cm^3^ s^−1^, for PBS rich blends a 4-step profile with higher velocities was used; holding pressure: 700–800 bar depending on composition; holding time 20 s (30 s PBS rich); cooling temperature and time: 30 °C/30 s. The compounds were pre-dried in a dryer (dry air generator, model: LUXOR 80; drying bins: 15 L, Motan-Colortronic GmbH, Kirchlengern, Germany) at 60 °C for 1.5 h immediately before injection molding.

Test specimens for fracture testing were prepared from the middle part of the tensile test bars (ISO 20753:2017).

The Young’s modulus (YM), tensile strength (σ) and elongation at break (ε) were measured according to DIN EN ISO 527-2 with a Zwick materials testing machine (type 1474 Retroline, Zwick GmbH & Co. KG, Ulm, Germany). The samples were tested under mono-axial tensile stress at a velocity of 50 mm min^−1^ (σ, ε) and 1 mm min^−1^ (YM) with a load cell of 50 kN. YM was measured between 0.05 and 0.25% strain. Test Expert III Software was used for calculation of the characteristic tensile test data. An instrumented Instron 9050 machine (Instron GmbH, Darmstadt, Germany) was used for Charpy-fracture tests according to ISO 179-1 and -2. A 5 Joule or 1 Joule pendulum with a maximum striker velocity of 2.9 m s^−1^ and a maximum deflection of 150° was used for instrumented un-notched or non-instrumented notched tests, respectively (specimen dimensions B = 10 mm, H = 4 mm, L = 80 mm, notch depth of 2 mm, notch radius rN = 0.25 ± 0.05 mm, edgewise). Instron Software CEAST View Version 6.20.2B was used to calculate the instrumented or non-instrumented elasticity (kJ m^−2^).

Samples were stored at 23 °C and 50% for 24 h prior to testing to meet standard requirements. The arithmetic mean and standard deviation of five or six measurements were calculated.

### 2.4. Thermal Analysis

Thermal properties were investigated by means of differential scanning calorimetry (DSC) and thermogravimetry (TG). A DSC 204 F1 Phoenix^®^ equipped with an automatic sample changer and liquid nitrogen cooling system and a TG F209 IRIS^®^ instrument were used (both: Netzsch Gerätebau GmbH, Selb, Germany). DSC experiments were conducted from room temperature up to 190 °C, down to −60 °C, and up to 190 °C again with a heating and cooling rate of 20 K min^−1^ and isotherms of 3 min at −60 °C and 190 °C. Characteristic thermal transition parameters were obtained from the first and second heating cycle. TG measurements were performed from 25 to 550 °C with a heating rate of 10 K min^−1^.

Analysis was performed with the Netzsch Proteus^®^ software for thermal analysis, version 7.

### 2.5. Scanning Electron Microscopy

Scanning electron microscopy (SEM) was performed in Vega3 Tescan (TESCAN ORSAY HOLDING a.s., Czech Republic) equipment using SE and BSE detectors and tungsten 20 kV excitation. Dog bone-shaped test specimens were cryogenically fractured using liquid nitrogen. SBP was used as received. All samples were sputter-coated with gold prior to examination. 

### 2.6. Particle Size

Particle size was measured with a laser diffraction particle analyzer (Mastersizer 2000, Malvern Panalytical GmbH, Kassel, Germany) equipped with a dispersing module Hydro 2000 G. Due to high water sorption capacity, the SBP was suspended in isopropanol and analyzed quickly. The general-purpose model with uneven shape was used for analysis of the data.

## 3. Results

### 3.1. Sugar Beet Pulp

**Figure 2:**
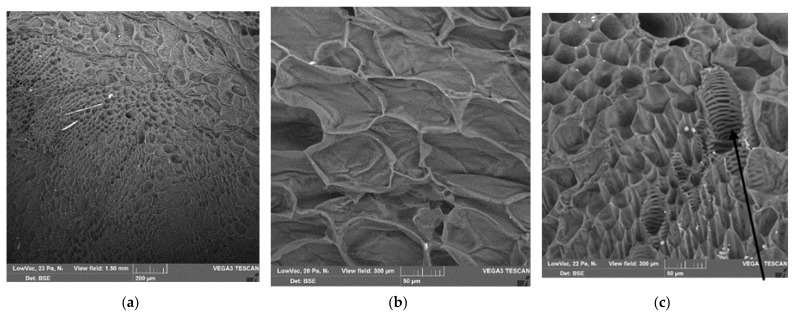
SEM images of freshly prepared sugar beet; (**a**) showing all sorts of cells (scale bar = 200 µm); (**b**) showing the sugar-containing parenchymal cells (scale bar = 50 µm); (**c**) showing the tubular xylem cells (arrow, scale bar = 50 µm); see text.

Figure 2 shows SEM micrographs of freshly prepared sugar beet. Two thirds of all cells of the beet are sugar-containing parenchymal cells with a diameter of 40–60 µm. The channels formed by the dead xylem cells (20–40 µm) are responsible for the transport of water and salts and are incrusted with lignin [3].

Due to the highly heterogeneous cell structure of the sugar beet, the degree of grinding may play an important role in SBP composites. In the literature, sizes of 30 µm [25] and 300 µm [29,30] are mentioned as well as sieve sizes of 300 mesh (~63 µm) [32] or 80 mesh (~180 µm) [34]. Our SBP types, ground by an air turbulence mill of type “Ultra-Rotor” as used in grain flour milling and sieved with a 30 µm or 450 µm classifier, have particle sizes of about 26, 529 and 645 µm (volume median, fine and coarse SBP, Table 1). Due to the natural origin of the SBP, the particle size distribution may vary from batch to batch to some extent. Thereby, the specific surface may be subjected to greater variations, as shown in Table 1 for two different coarse type batches (I and II).

**Figure 3:**
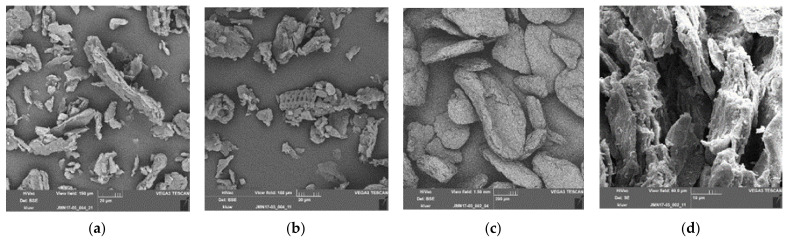
SEM images of ground SBP; (**a**,**b**): scale bar 20 µm; SBP ground to about 23 µm (d50) showing only fragments of cell walls; from (**a**) thicknesses of about 2 µm of the cell wall can be extracted; in (**b**) a part of a crushed incrusted xylem cell can clearly be seen; (**c**,**d**): SBP ground to approximately 600 µm (d50) showing compacted ellipsoidal-shaped particles (**c**, scale bar 200 µm); in (**d**) delamination of a particle is shown in higher resolution (scale bar 10 µm).

As can be seen from Figure 3, the shape of SBP may be described as unevenly shaped particles, as platelets (Figure 3a,b) or as ellipsoidal-shaped particles (Figure 3c), depending on the fineness of the SBP.

### 3.2. Thermoplasticization of Sugar Beet Pulp (TSBP)

As mentioned in the literature by Rouilly et al. [2,23,24], SBP can be plasticized using an adequate screw design if sufficient specific mechanical energy (SME) is applied to the system. Following Rouilly et al., SME values of about 500 Whkg^−1^ should be applied [2].

**Figure 4:**
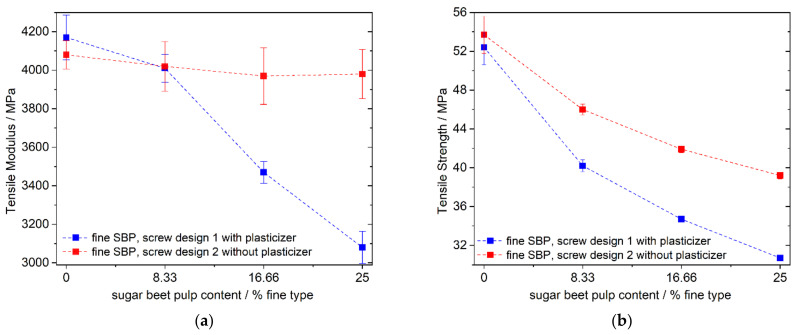
(**a**) Tensile modulus and (**b**) tensile strength of PLA–PBS (9:1)–SBP–chalk composites (75% PLA–PBS) using a compound produced with screw design 1 or 2.

We found that our screw design 1 was able to produce thermoplasticized SBP with different amounts of water (45 or 55%) or glycerol (32 or 45%). The specific mechanical energy input was higher with glycerol than with water and higher with 45% of water than with 55% of water. SME was also higher with a higher rotation speed. Specific mechanical energy uptake of about 1300 Whkg^−1^ was reached with both the fine and the coarse material by applying 250 rpm and 45% water. By applying a lower rotational speed or/and more water, SME input can be adjusted to lower values with a minimum of about 400 or 500 Whkg^−1^ for the fine or coarse SBP. Nevertheless, we decided not to use pre-plasticized SBP because (1) this step causes additional costs and energy consumption and (2) a blocking of the extruder in the second mixing zone sometimes occurred. We attribute this blocking to the susceptibility of the screw design to dosage inhomogeneity leading to higher temperature and burning in the second mixing zone. Embedding the SBP in the plastic matrix should prevent the burning of the SBP and blocking of the extruder in the second mixing zone. Therefore, we tested screw design 1 for producing a PLA–PBS–TSBP (glycerol/SBP = 0.32:1) composite in one step. As a reference, we used a cost-effective composite with 75 mass percent of PLA–PBS matrix (9:1 wt./wt.) and 25 mass percent chalk as filler (see also Section 3.3) and replaced the chalk in three steps with SBP. Mechanical and thermal analysis data showed that the glycerol was not completed absorbed by the SBP because of strong modulus depression (Figure 4a) and a slight reduction in the glass transition temperature (Table 2) of the compound. Parts of the glycerol must have plasticized the PLA phase as a side effect. In comparison, the same compounds produced with a gentler screw design (screw design 2) and without the addition of the plasticizer show higher mechanical values. The plasticizer was intentionally not added since the gentler screw design 2 does not lead to improved incorporation of the plasticizer into the SBP, and the transesterification of the polyester matrix by the plasticizer can occur during compounding and in the long term. In comparison to the chalk-filled reference, the tensile modulus remains nearly constant (Figure 4a) and the tensile strength is somewhat reduced (Figure 4b), which is expected when replacing chalk with a low-modulus, highly polar filler.

Furthermore, the plasticization of the matrix with glycerol is not recommended because of the migration of the low molecular weight plasticizer, which can change the morphology and can possibly lead to long-term degradation of the molecular weight of the polyester by transesterification.

### 3.3. Composites Based on PLA, PBS and SBP

As a consequence of the results from Section 3.2 and the dimensions of our fine SBP type, which showed destruction of the sugar beet pulp below cell dimensions, we decided to produce SBP-containing composites using screw design 2 and (with lower energy input, shear and residence time) without the addition of a plasticization agent for SBP. In practice, a wide range of PLA–PBS compositions with different filler materials are used. Therefore, we decided to investigate three different polymer matrix formulations regarding the use of SBP as a filler.

#### 3.3.1. High PLA Content

A reference composition with a high PLA content was formulated with a filler loading of 25 percent chalk and 75 percent of a nine to one mixture of PLA and PBS (overall composition: PLA–PBS–chalk = 67.5:7.5:25). We replaced the mineral filler by SBP in three steps. In addition, we varied the amount of a maleic anhydride-modified PLA coupling agent. From a rule of thumb in natural fiber-based composites, we chose a starting level of one percent coupling agent per 10 percent natural fiber.

**Figure 5:**
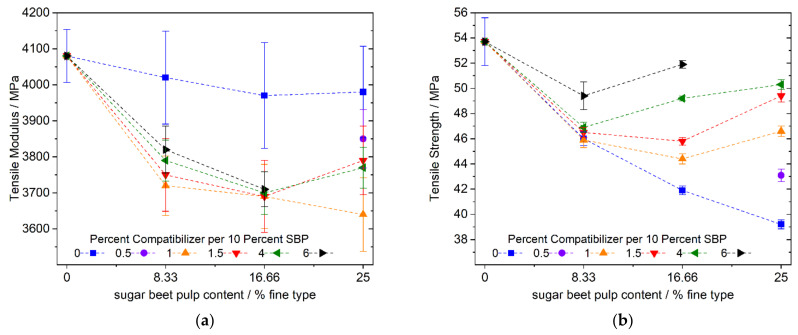
Tensile modulus and strength of PLA–PBS (9:1)–SBP composites with 8.33, 16.66 and 25% fine SBP type and 1.0, 1.5, 4 and 6% compatibilizer per 10% SBP; (**a**): tensile modulus, (**b**): tensile strength; instead of a sample with 25% SBP and 6% compatibilizer per 10% SBP, a sample with 0.5% compatibilizer SBP per 10% SBP was used.

Replacing the mineral filler with fine SBP leads, as expected, to a reduced tensile modulus of elasticity (Figure 4a) due to the weaker inherent stiffness of the SBP in contrast to chalk. Surprisingly, however, for fine SBP and no added compatibilizer (Figure 5a), blue data points), this decrease is small and almost negligible considering the standard deviation. We suspect that another process is responsible for compensating the expected reduction in modulus caused by changing from chalk to SBP.

One possible reason leading to a higher modulus in a PLA matrix is an increase in crystallinity. According to Aliotta et al. [38], the amorphous PLA phase contributes 3200 MPa and the crystalline phase contributes 8500 MPa to the modulus. Increasing the crystallinity of the PLA phase in our composites by one percent will therefore raise the tensile modulus by ~35 MPa. Regardless of the amount of SBP and the amount of compatibilizer, the moduli of all the samples containing SBP and a compatibilizer are in the range of ~3650–3850 MPa. Considering the standard deviations, the use of SBP with a compatibilizer leads to an average decrease in the modulus of elasticity of ~8.5 percent (∅~3740/4080) (Figure 5a) compared to the samples with SBP without a compatibilizer. The expected higher stiffness of the composite by using a compatibilizer for improving the SBP–polymer matrix interface must have been counteracted by morphological changes, e.g., less crystallinity. Nevertheless, the good effectiveness of the coupling agent is shown by the dependence of the tensile strength on the amount of used compatibilizer (Figure 5b): the strength of uncompatibilized composites is reduced by up to 27 percent at 25% SBP content. Due to the strong difference in their polarities, there is only weak transmission of forces between the components without a compatibilizer. As expected, the strength increases when the compatibilizer is added. Upon adding larger quantities, the SBP composite almost reaches the initial strength of the chalk-filled PLA–PBS blend. However, because of the highly polar nature and the high surface area of the fine type of the SBP in comparison with commonly used natural fibers, amounts of up to 6 percent coupling agent per 10 percent SBP are required, which is not economically justifiable.

**Figure 6:**
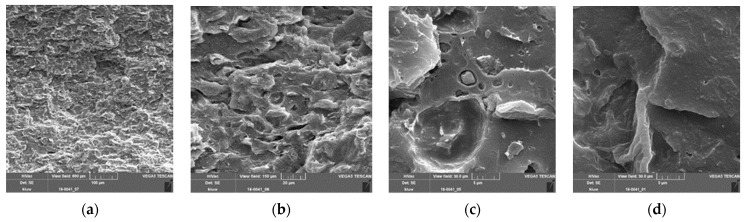
SEM images of PLA–PBS (9:1)–SBP composites, fine SBP type, 25%, 3.75% coupling agent (=1.5% per 10% SBP); (**a**) scale bar = 100 µm; (**b**) scale bar = 20 µm, magnification of (**a**), holes caused by extracted particles and surfaces caused by splintered particles can be seen; well-bonded particles are also present, cracks in the matrix above the well-bonded particles indicate strong adhesion of this particle to the matrix; (**c**) scale bar 5 µm, magnification of (**b**); (**d**) scale bar = 5 µm, a very well-bonded particle is shown; in the matrix, no PBS phase can clearly be seen, indicating good compatibility of the PLA and PBS phases [37].

Figure 6 shows some cryogen-fractured surfaces of the PLA–PBS–SBP composite with 25 percent fine SBP. Figure 6a shows an overview of the fracture surface, which does not show any specific fracture boundaries and appears homogeneous in itself. In the magnifications in Figure 6b,c, holes of pulled-out SBP as well as well-bonded SBP particles can be seen. Additionally, a surface that may be caused by a delaminated particle of SBP is shown in Figure 6c. In Figure 6d, a very well-integrated SBP particle is shown.

**Figure 7:**
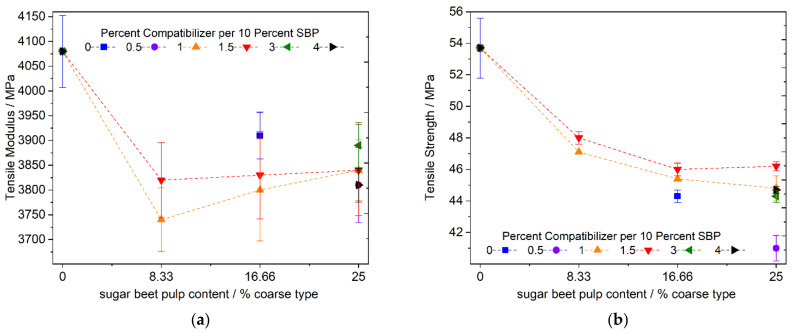
Tensile modulus and strength of PLA–PBS (9:1)–SBP composites with 8.33, 16.66 and 25% coarse SBP and 1.0 and 1.5 compatibilizer per 10% SBP; (**a**): tensile modulus, (**b**): tensile strength; the influence of higher and lower amounts of compatibilizer was checked for the samples with 25% SBP.

The use of compatibilized coarse SBP results in a similar picture for the modulus of elasticity and tensile strength (Figure 7a,b). The modulus is lowered by adding SBP for chalk with no effect of the compatibilizer, considering the standard deviations. In addition, the strength is lowered and almost no dependence on the amount of compatibilizer can be seen. Higher amounts of coupling agent (e.g., 3 or 4% per 10% of SBP, Figure 7b) even lead to lower values.

However, regarding the strength, a small effect of the compatibilizer can be seen by comparing the values when using no (or 0.5%) compatibilizer per 10% SBP with the ones for higher compatibilizer addition (1.0 or 1.5).

**Figure 8:**
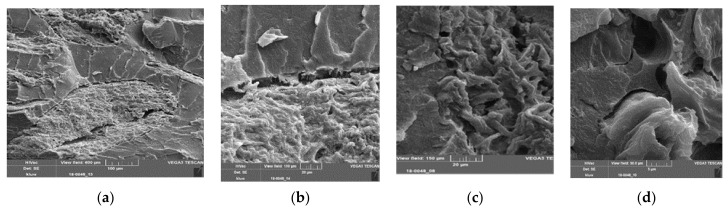
SEM images of PLA–PBS (9:1)–SBP composites, coarse SBP type, 25% and 3.75% compatibilizer (=1.5% per 10% SBP); (**a**) scale bar = 100 µm, a crack through a particle and delamination are shown; (**b**) scale bar = 20 µm, magnification of (**a**); (**c**) scale bar = 20 µm, delamination within a particle can be assumed but the particle has also good adhesion to the matrix; (**d**) scale bar = 5 µm, a hole from a pulled out subcellular particle and partly de-bonded particles can be seen.

Nevertheless, a plateau value in the range of 46 MPa seems to be achievable, but there is no strong dependency of the strength on the amount of compatibilizer, as in the case of the fine SBP type. This behavior of the strength of the composite may be due to the internal strength of the coarse SBP particle, since SEM images of fracture patterns of cryogenically broken test specimens show cracks in the big SBP particle (Figure 8a). Therefore, the inherent weakness of compressed cell-scale fragments of SBP is responsible for the reduced strength compared to the fine SBP type with subcellular particles.

The usage of a coupling agent type compatibilizer, anchoring one phase to the other by chemical bonds and chain entanglement, is one way to improve the stiffness and strength of a composite. Since the phases in a composite must be close to each other at the molecular level, the wettability of one phase by the other is crucial. Due to the high content of beta-glycosides and glucuronic acids in SBP (cellulose + hemicellulose + pectin), the surface may be covered with hydroxyl, ester and carboxyl groups, resulting in a highly polar surface with solubility parameters greater than the 29 or 36 J0.5cm1.5 as in methylated, hydroxypropylated or pure cellulose [39,40]. Therefore, a modification of the surface by a reactive monomer changing the surface energy and promoting adhesion is another way of compatibilization [41]. PLA and PBS are mid-polar polyesters with solubility parameters of about 21 to 22 J0.5cm1.5 [37,42] using the Hoftyzer–van Krevelen or Hansen three-dimensional model. Using the one-dimensional Hildebrandt model with summarized data from several authors from the book of Robeson [41], the parameters are about 18.8 to 20.4 J0.5cm1.5 for the glassy state of PLA and 18.3 to 19.9 J0.5cm1.5 for the rubbery state of PBS. Covering the surface of SBP with mid-polar molecules to reduce its polarity closer to that of the polymer components should therefore improve wettability. A commercially available reagent is 1,2,3-propanetriol-glycidyl-ether. This reactive monomer of epoxide-type can react with the acid groups of the pectin or the hydroxyl groups of the cellulose at an elevated temperature in short times.

**Figure 9:**
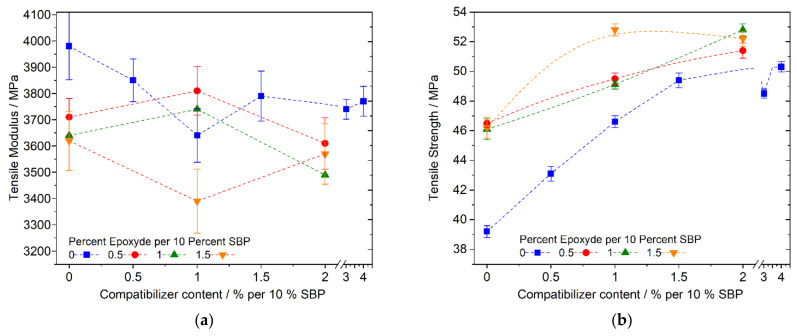
Dependence of tensile modulus (**a**) or strength (**b**) of PLA–PBS (9:1)–SBP composites (25% SBP) on the addition of reactive adhesion promoter (epoxide) and coupling agent (PLA-g-MAH).

Figure 9b shows that the surface modification of the fine SBP type by using 1,2,3-propanetriol-glycidyl-ether has a great effect on the strength. Only 0.5 percent per 10 percent of SBP improves the strength by 18 percent, even without the addition of the PLA-based compatibilizer. The further increase in tensile strength with the addition of more epoxide is less pronounced and not completely uniform (1% compatibilizer, 1.5% epoxide). The trend of the data points suggests a maximum tensile strength value in the range of 50–52 MPa. No effect of the addition of the glycidyl ether on the modulus can be seen, taking standard deviations into account (Figure 9a). In the case of the coarse SBP type, the influence of the adhesion promoter is significantly lower. The strengths of the compounds with 25% SBP and no chalk do not exceed 45 MPa by adding 1% coupling agent and 0.5, 1 or 1.5% adhesion promoter, and the data can also be explained in part by standard deviation (see Table 3).

Chen et al. [31] reported a similar effect when treating a PLA–SBP composite with pMDI (polymeric methylene diphenyl diisocyanate). The strength of a 7:3 composite of PLA and SBP increased from 37 to 50–60 MPa when adding 0.5 to 3% pMDI, with the highest value occurring with the application of 2% pMDI. As they mixed the components before extrusion, we believe that the absorbed highly reactive pMDI reacted preferably with the carboxyl and hydroxyl groups at the surfaces of the SBP (a 30 µm-sized type). From our findings, we conclude that in their experimental set-up pMDI acted as an adhesion promoter or surface modification agent and did not behave as a coupling agent, anchoring the phases together, as claimed in their paper.

Taking into account the respective costs of the compatibilizers used and the application-specific requirements for the strength of PLA–PBS–SBP composites, cost optimal composition can develop.

All of the PLA–PBS (9:1)–SBP composites are characterized by brittle fracture failure (Table 3). The impact strength tends to increase with a lower SBP content. It decreases with an increasing content of fine SBP and is generally poor with coarse SBP, irrespective of the compatibilizer content. All materials possess low notched impact strengths. The elongation at break remains on the level of the reference, with slight improvements upon adding specific impact modifiers (Table 4).

The addition of a polyolefinic impact modifier (Acti-Tech) improves the impact behavior and the elongation at break only insignificantly when higher amounts are used. However, this is at the expense of the other mechanical characteristics: the modulus decreases by approx. 1 GPa, and the tensile strength by pprox. 20 Mpa. The core-shell-type impact modifier (Biostrength^®^) slightly increases the unnotched impact resistance, but does not affect the elongation at break. From acoustic emission analysis, Finkenstadt et al. [30] deduced that the de-bonding of phases takes place before rupture. Therefore, the fracture mechanics may be determined by the weakest structures of the SBP and its interfaces.

#### 3.3.2. Medium and High PBS Content (PBS Rich Compound)

Choosing a higher PBS content will soften the composite and will make the composite more easily degradable at ambient and slightly elevated temperatures as in composts. In addition, in many commercialized PBS compounds, chalk is replaced by talc. Therefore, we formulated compositions with talc and less filler content due to the higher price and the higher modulus of talc. A filler content of 16% talc and 84% of a four to three or three to four mixture of PLA and PBS (PLA:PBS:talc = 48:36:16 or 36:48:16) was chosen. We replaced the mineral filler by SBP in two steps.

**Figure 10:**
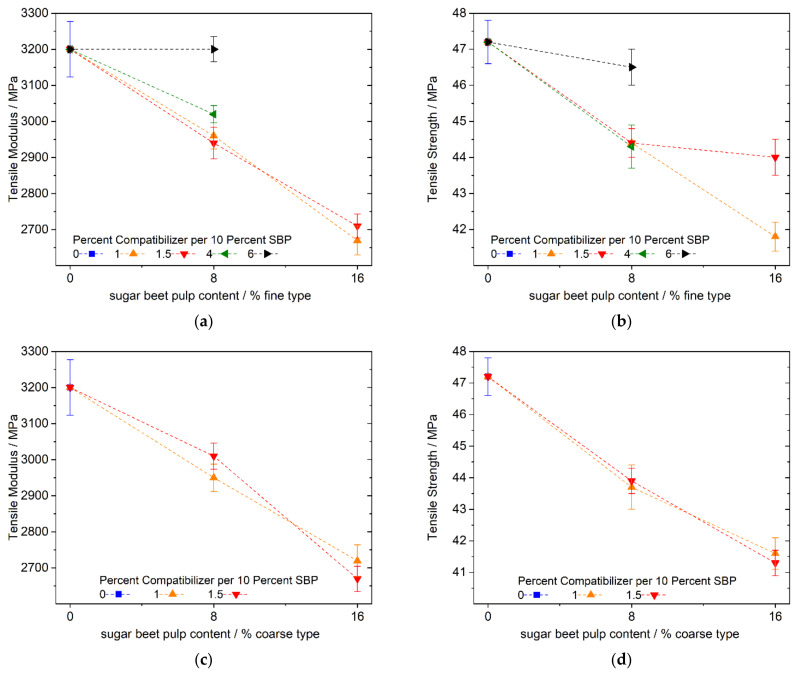
Tensile modulus and strength of PLA–PBS (4:3)–SBP composites; fine type: (**a**) modulus, (**b**) strength; coarse type: (**c**) modulus, (**d**) strength.

**Figure 11:**
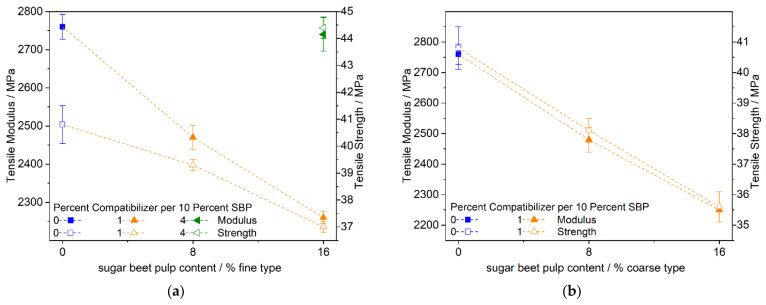
Tensile modulus and strength of PLA–PBS (3:4)–SBP composites: (**a**) fine type, (**b**) coarse type.

Figure 10 and Figure 11 show the tensile properties of the PLA–PBS (4:3) and (3:4) composites, respectively, with 0, 8, and 16 percent SBP. In contrast to the PLA–PBS (9:1) composites, the modulus and strength decrease almost linearly with higher amounts of SBP using the fine or coarse type of SBP and small amounts of the compatibilizer. For the composites with the fine SBP type, we tested the influence of higher amounts of the compatibilizer exemplarily. Even though the compatibilizer is based on PLA (PLA-g-MAH; to our knowledge no commercialized PBS-g-MAH is available yet), the compatibilizer also works in PBS-rich composites.

These surprising findings may be due to the following facts: the PLA-g-MAH compatibilizer agent should mainly be solved in the PLA phase due to the poor miscibility of PLA and PBS [37]. As an example, the relative amounts (mass-based) of PLA-g-MAH in the PLA phase in the composites with higher PBS amounts (PLA–PBS = (4:3) or (3:4), respectively) with 8% SBP and 0.8% PLA-g-MAH or 16% SBP and 1.6% PLA-g-MAH, respectively (Figure 9a and Figure 10a), are 1.7 and 4.4%. In the PLA–PBS (9:1)–SBP composites with 8.33 or 16.66% SBP and 0.83 or 1.66% PLA-g-MAH, the relative amounts are 1.2 and 2.5%, respectively. Assuming a PLA coating of the surfaces of SBP in the PLA–PBS = (4:3) or (3:4) composites, the improvement in the modulus and strength at higher amounts of compatibilizer can be explained. Furthermore, the transesterification of PBS with PLA-g-PLA may take place at the higher amount of interfaces in the PLA–PBS = (4:3) or (3:4) composites during compounding, leading to a better compatibility of the polymer phases to some extent.

In contrast to the PLA-rich PLA–PBS (9:1)–SBP composites, an influence of the SBP content on the crystallization of the PLA–PBS (4:3)–SBP composites can be seen. While the SBP-free composites and the composites with 8% talc and 8% SBP crystallize almost completely from the melt upon cooling with 20 Kmin^−1^, samples without talc and with 16% SBP show significant post-crystallization enthalpies in the range of 20–25% of the sum of the melt enthalpies of PBS and PLA. In PLA–PBS (3:4)–SBP composites, this denucleating effect is only seen to a much more limited extent in the sample with 16% fine SBP and without talc.

The nucleating effect of talc on PLA is well known, and Pivsa-Art Y et al. report that in PLA–PBS (8:2/6:4)–talc composites, the crystallization of PLA is promoted and the crystallization of PBS is inhibited [43,44,45]. Thus, in talc-filled PLA–PBS (4:3)–SBP composites, the replacement of talc by SBP has a denucleating effect. In the talc-free PLA–PBS (3:4)–SBP composites, the denucleating effect of talc on the PBS main phase is outweighed in particular by finely ground SBP. It can be deduced that the chalk and SBP-filled PLA-rich PLA–PBS (9:1)–SBP composites can be adjusted in their thermal mechanical properties to some extent by adding talc, if necessary.

Mechanical data of the PLA–PBS (4:3) and (3:4) composites are summarized in Table 5. The tensile modulus and strength are reduced in comparison to the PLA-rich composites, as expected since PBS is a softer material. As in the case of the PLA–PBS (9:1)–SBP composites, the notched impact strength is poor for these materials. Due to the higher amount of soft PBS, the elongation at break is somewhat enhanced on average. However, the absolute values still are very low. Requirements from real applications must show if the strength of the composite is high enough for accepting elongation at break in the single-digit range.

**Figure 12:**
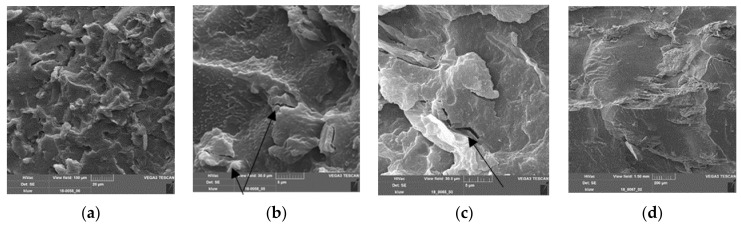
(**a**,**b**) SEM images of PLA–PBS (4:3)–SBP composites, 16% fine SBP type, 1.5% compatibilizer/10% SBP (scale bar = 20 and 5 µm); black arrows: cracks in a SBP particle, white arrow: crack at the interface; (**c**) SEM images of PLA–PBS (3:4)–SBP composites, 16% fine SBP type 1% compatibilizer/10% SBP (scale bar = 5 µm), white arrow: crack at the interface, black arrow: crack in the polymer matrix; (**d**) SEM images of PLA–PBS (3:4)–SBP composites, 16% coarse SBP type, 1% compatibilizer/10% SBP (scale bar = 200 m), the flat area may be caused by detached SBP particles.

In Figure 12a,b, images of cryogenically fractured samples of PLA–PBS (4:3) composites with 16% SBP are shown. Figure 12a shows many particle pull-outs and a rough fracture surface. In Figure 12b, cracks through SBP particles and at the interface are clearly visible.

Figure 12c,d show images of cryogenically fractured samples of compounds with the highest PBS content (PLA–PBS (3:4)). A crack in the matrix phase near the SBP–matrix interface can clearly be seen (Figure 12c). The fracture pattern in Figure 12d with well-bonded coarse SBP particles, but also some delamination inside the particles, resembles a delaminated surface (conchoidal fracture).

### 3.4. Influence of SBP on Modulus and Strength: Theoretical Considerations for the Mineral Filler-Free Composites

The simplest approach to predicting composite properties is to check if the dispersed phase can be considered as a non-interacting phase (equivalent to a void) or not (Equation (2)) and then use the rule of mixtures (Equation (3), left part) [46,47,48,49,50,51] for calculating the upper limit. For this, only the respective characteristic values of the components are required.

(2) $$E_{c}=\left(1-\phi_{SBP}^{2/3}\right)\times E_{m}$$

(3) $$E_{c}=\underset{i}{\sum }E_{i}\times \Phi_{i}↔\;E_{j}=\left(E_{b}-\underset{i\neq j}{\sum }E_{i}\times \Phi_{i}\right)/\Phi_{j}$$

Conversely, if one characteristic value is unknown, it can be estimated from measurements on the composite (Equation (3), right side), where *E_c_*, *E_i_*, *E_j_* and *E_m_* are the moduli of the composite, the component *i* and *j* or the modulus of the matrix, respectively, and *Φ_i_*, *Φ_j_* and *Φ_SBP_* are the volume fractions of the component *i*, *j* or *SBP*. This approach has the advantage that deviations from the idealized upper limit (e.g., caused by non-optimal interfaces) can be assigned to the filler and the process conditions. In particular, the compatibilization can be evaluated.

A modulus of about 2000–2100, 1600–1700 and 1300–1400 MPa can be expected for the composites with 25% SBP and PLA: PBS = 9:1 or 16% SBP and PLA: PBS = 4:3 or 3:4, respectively, by using Equation (2) when no interaction between the polymeric matrix and the sugar beet pulp takes place. Common values for the modulus of PLA and PBS [37] of 3500 and 630 MPa, respectively, and densities of PLA and PBS of about 1.24 and 1.26 g cm^−3^ (from actual brochures from the producers) and 1.4–1.5 gcm^−3^ for the SBP (estimated) were used. The matrix modulus of the PLA–PBS matrix was thereby calculated using Equation (3) assuming good interaction for PLA and PBS. The measured values are higher than those numbers and, therefore, some interactions between SBP and the polymeric matrix take place.

The modulus is measured at very small elongations where the influence of the fiber matrix adhesion is not yet as pronounced as when determining the tensile strength. Furthermore, the modulus is also influenced by the processing conditions and the semi-crystalline morphology formed from them. We have thus calculated one rough guide value from the data in Table 3 and Table 4 using only the data of the mineral-free samples and applying the root mean square approach for minimizing the differences of the calculated and the measured values. In this estimate, morphologic changes due to the variation in matrix components and due to the use of compatibilizers are added to the modulus of the SBP. When looking at individual values, they vary from about 4000 to 7700 MPa for the fine type and 4400 to 7300 for the coarse type. A mean modulus of about 5100 MPa for the fine type and 5700 for the coarse type can be estimated. The slightly lower value for the fine type may be explained by the 25 to 50-fold higher specific surface (see Table 1) and the more particular shape, having a lower aspect ratio, of the fine type. The best enhancement in modulus is achieved with the composites highest in PBS content and compatibilized with the PLA-g-MAH and the epoxy-type adhesion promoter. Using the only available literature data from Chen et al. [31], who used polymeric methylene diisocyanate as an adhesion promoter, we extracted a modulus of about 7000 MPa from their data. In conclusion, in terms of modulus, SBP behaves more like a filler than a stiffener. With careful optimization of the processing conditions (including compatibilization), a noticeably increased modulus may be reached. However, even the simplest mineral filler, chalk, which can also keep up economically with SBP, is capable of better performance.

Tensile strength is much more sensitive to the quality of compatibilization due to the higher strains at tensile strength and thus the necessary ability of the plastic matrix to yield. Otherwise, phase breakage and rapid fracture occur. The often-used model for describing the composite strength is the Nicolais–Narkis equation (Equation (4)):

(4) $$\sigma_{c}=\left(1-K\phi_{SBP}^{2/3}\right)\times \sigma_{m}$$

where *σ_c_* and *σ_m_* are the strengths of the composite and the matrix, respectively, *ϕ_SBP_* is the volume fraction of *SBP* and *K* is an adjustable interphase interaction parameter between 0 and 1.21 [48,49,52,53]. *A* low value of *K* indicates good compatibility. Table 6 summarizes the calculated values for *K*.

It is clearly visible that the coarse SBP type exhibits K-values greater than about 0.5 and that even high amounts of compatibilizer do not lead to lower values, indicating the inherent weakness of coarse SBP particles, as discussed in the previous sections. In contrast, sufficient additivation of the fine SBP type can significantly improve its binding to the matrix, which can be deduced from low values of K with small amounts of compatibilizers. Nevertheless, the non-uniform trends of the K-values of the fine SBP type, which may be caused by the lab-scale trials with manual steps for incorporating the adhesion promoter, indicate that the processing will have an influence on the strength.

## 4. Conclusions

Sugar beet pulp (SBP) is a residue available in high amounts from the sugar industry that can act as a cheap bio-based and biodegradable filler for fully bio-based compounds based on bio-based polyesters. The more cubic, spherical or ellipsoidal-shaped particles of SBP ground by an air turbulence mill of type “Ultra-Rotor” are not suitable for usage as a fiber-like reinforcement. Furthermore, the fineness of ground SBP has an influence on the properties due to (i) the high amount on polar surfaces, leading to insufficient compatibility, and ii) due to the inner structure of the particulate matter, limiting the strength of the composite to the cohesive strength of compressed sugar-cell compartments of the SBP. The compatibilization of the interface between the polymer matrix and SBP can be achieved by using a compatibilizer with different modes of operations. Compatibilization is limited by the inherent weakness of large SBP particles or by the high surface area of fine particles, which results in an economically unjustifiable quantity of compatibilizer needed. SEM fracture patterns show that compatibilization can lead to well-bonded particles with a cohesive fracture pattern in the matrix besides the SBP–polymer matrix interface. When producing composites from SBP and bio-based polyesters, attention should be paid to the optimal incorporation of compatibilizers and blend quality. Nevertheless, SBP composites are not reinforced materials, but materials additivated with cost-effective biodegradable filler. The selection of the compatibilizer should therefore be made based on the appropriate raw materials available locally, taking into account toxicological, safety and cost considerations and the requirements for the material.

## 5. Outlook

Due to its high pectin content, SBP has a high water absorption capacity compared to other fillers or classic agricultural fibers. The water absorption kinetics of SBP-filled bio-based polyesters will be discussed in part two of this article.

## Figures and Tables

**Table 1:** Characteristic sizes of fine and coarse SBP types.

Dimension	Unit	Fine Type	Coarse Type (I)	Coarse Type (II)
d (0.1)	µm	6.1	360	291
d (0.5)	µm	23.4	601	500
d (0.9)	µm	48.7	999	821
surface mean value D[3, 2], Sauter-diameter	µm	11.8	539	280
volume mean value D[4, 3], De Broucker-diameter	µm	25.9	645	529
specific surface	cm^2^ g^−1^	5080	111	214

**Table 2:** Glass transition temperatures (2nd heating curve, 20 Kmin^−1^) of the PLA phase.

Sample, Consisting of 75% PLA–PBS (9:1) and	Screw Design 1, Glycerol-Containing Composite T_g_/°C	Screw Design 2, Composite without Glycerol T_g_/°C
0% SBP 25% chalk	54.7	
8.33% SBP 16.66% chalk	53.5	54.7
16.66% SBP 8.33% chalk	50.5	56.3
25% SBP 0% chalk	50.9	52.4

**Table 3:** Mechanical data of PLA–PBS (9:1)–SBP composites; relative standard deviation in brackets is given in percent of the mean value; ^1^ f = fine, c = coarse, ^2^ c. a. = coupling agent, a. p. = adhesion promoter, both added in given percentage per 10% SBP; n.d. = not determined.

SBP		Chalk	Additives		Tensile Modulus	Tensile Strength	Elongation at Break	Impact (Charpy)	
Type ^1^	%		c. a. ^2^	a. p. ^2^	MPa	MPa	%	Notched kJm^−2^	Unnotched kJm^−2^
-	0	25	0	0	4080 (1.8)	50.4 (1.0)	3.0 (8.2)	1.6 (0.8)	14.2 (7.2)
f	8.3	16.7	0	0	4020 (32)	46.0 (1.2)	4.1 (14)	2.4 (25)	17.6 (8.5)
f	8.3	16.7	1	0	3720 (2.2)	45.9 (1.4)	5.0 (22)	2.0 (12)	18.9 (7.8)
f	8.3	16.7	1.5	0	3750 (2.7)	46.5 (0.9)	4.2 (27)	2.0 (8.0)	17.5 (13)
f	8.3	16.7	4	0	3790 (1.5)	46.9 (0.8)	4.2 (8.8)	1.8 (6.1)	19.4 (5.9)
f	8.3	16.7	6	0	3820 (1.7)	49.4 (2.3)	3.8 (17)	2.0 (8.7)	17.3 (2.3)
f	16.7	8.3	0	0	3970 (3.7)	41.9 (0.8)	3.8 (16)	2.5 (22)	13.3 (9.2)
f	16.7	8.3	1	0	3690 (2.4)	44.4 (0.9)	3.8 (15)	1.9 (13)	15.5 (16)
f	16.7	8.3	1.5	0	3690 (2.7)	45.8 (0.6)	3.5 (8.7)	2.1 (13)	15.3 (6.6)
f	16.7	8.3	4	0	3700 (1.6)	49.2 (0.49	3.7 (1.0)	1.8 (9.9)	14.9 (19)
f	16.7	8.3	6	0	3710 (1.3)	51.9 (0.5)	3.8 (3.2)	1.8 (7.5)	16.6 (4.0)
f	25	0	0	0	3980 (3.2)	39.2 (0.9)	3.9 (6.0)	2.1 (18)	13.5 (6.5)
f	25	0	0	0.5	3710 (1.9)	46.6 (0.7)	3.2 (5.5)	1.7 (4.4)	12.4 (6.2)
f	25	0	0	1	3640 (5.7)	46.1 (1.5)	3.1 (9.2)	1.8 (8.1)	14.7 (9.3)
f	25	0	0	1.5	3620 (3.1)	46.2 (1.6)	2.9 (4.5)	1.6 (5.8)	14.1 (3.6)
f	25	0	0.5	0	3850 (2.1)	43.1 (1.1)	3.5 (16)	n.d.	14.9 (11)
f	25	0	1	0	3640 (2.8)	46.6 (0.9)	3.3 (6.5)	n.d.	14.1 (9.9)
f	25	0	1	0.5	3810 (2.4)	49.5 (0.8)	3.3 (4.6)	1.6 (8.5)	12.8 (6.2)
f	25	0	1	1	3740 (1.4)	49.1 (0.7)	3.2 (3.4)	1.8 (6.6)	13.3 (4.2)
f	25	0	1	1.5	3390 (3.6)	52.8 (0.7)	4.4 (8.7)	1.7 (6.8)	18.6 (7.8)
f	25	0	1.5	0	3790 (2.5)	49.4 (1.1)	3.3 (11)	n.d.	13.0 (9.8)
f	25	0	2	0.5	3610 (2.7)	51.4 (0.9)	3.4 (5.4)	1.7 (6.5)	8.6 (8.5)
f	25	0	2	1	3490 (4.5)	52.8 (0.7)	3.6 (3.3)	1.9 (17)	16.7 (5.1)
f	25	0	2	1.5	3570 (3.2)	52.2 (0.9)	3.8 (9.2)	1.7 (9.5)	18.4 (5.0)
f	25	0	3	0	3740 (1.0)	48.5 (0.6)	1.6 (6.2)	1.7 (4.8)	13.4 (7.3)
f	25	0	4	0	3770 (1.5)	50.3 (0.7)	3.3 (4.7)	1.8 (12)	14.2 (7.9)
c	8.3	16.7	1	0	3740 (1.7)	47.1 (0.8)	3.2 (4.1)	n.d,	8.2 (20)
c	8.3	16.7	1.5	0	3820 (2.0)	48.0 (0.8)	3.2 (5.5)	1.8 (10)	9.3 (6.7)
c	16.7	8.3	0	0	3910 (1.2)	44.3 (0.9)	2.9 (5.8)	2.3 (23)	8.8 (6.2)
c	16.7	8.3	1	0	3800 (2.7)	45.4 (0.8)	3.1 (5.0)	n.d	8.0 (16)
c	16.7	8.3	1.5	0	3830 (2.3)	46.0 (0.9)	3.1 (2.8)	n.d	8.6 (18)
c	25	0	0.5	0	3810 (2.0)	41.0 (1.9)	2.8 (4.3)	n.d	6.7 (15)
c	25	0	1	0	3840 (1.6)	44.8 (0.7)	2.9 (5.3)	n.d	8.6 (9.5)
c	25	0	1.5	0	3840 (2.4)	46.2 (1.3)	3.0 (1.5)	n.d	8.2 (9.1)
c	25	0	1	0.5	3780 (0.6)	42.5 (0.9)	3.0 (3.5)	1.9 (17)	14.2 (7.2)
c	25	0	0	1	3560 (1.7)	43.1 (1.0)	3.4 (4.1)	1.9 (14)	7.8 (16)
c	25	0	1	1	3520 (3.0)	44.7 (0.6)	3.2 (3.7)	1.8 (11)	8.5 (7.3)
c	25	0	1	1.5	3580 (3.0)	44.9 (2.3)	3.1 (5.6)	1.7 (7.1)	7.8 (20)
c	25	0	3	0	3890 (1.2)	44.3 (0.8)	3.0 (5.4)	1.8 (6.1)	8.9 (8.0)
c	25	0	4	0	3810 (0.9)	44.7 (0.5)	3.0 (1.7)	2.0 (22)	8.8 (3.2)

**Table 4:** Mechanical data of impact-modified PLA–PBS (9:1)–SBP (25%) composites; relative standard deviations (%) in brackets.

SBP	Impact Modifier		Tensile Modulus	Tensile Strength	Elongation at Break	Impact (Charpy)	
Type	Type	%	MPa	MPa	%	Notched kJm^−2^	Unnotched kJm^−2^
f	core-shell	3.75	3410 (1.2)	40.8 (0.8)	3.9 (3.8)	1.8 (9.1)	14.9 (8.3)
f	core-shell	7.5	3170 (1.9)	39.2 (1.7)	3.8 (5.4)	2.2 (20)	15.5 (5.0)
f	core-shell	11.25	2860 (0.8)	35.7 (0.5)	5.1 (21)	2.4 (12)	16.8 (12)
f	core-shell	15	2660 (1.9)	32.0 (2.8)	6.2 (13)	2.9 (5.2)	19.2 (7.7)
f	polyolefinic	3.75	2920 (2.6)	32.1 (1.0)	3.8 (8.7)	2.6 (16)	8.4 (4.9)
f	polyolefinic	7.5	2390 (1.9)	29.0 (1.1)	9.7 (8.3)	n.d.	18.4 (17)
c	polyolefinic	3.75	2810 (1.0)	31.0 (1.3)	4.3 (7.6)	3.0 (7.4)	8.2 (3.3)
c	polyolefinic	7.5	2790 (0.9)	31.8 (0.6)	8.8 (9.1)	2.3 (14)	14.8 (12)

**Table 5:** Mechanical data of PLA–PBS (4:3) and (3:4)–SBP composites; relative standard deviations (%) in brackets; ^1^ f = fine, c = coarse, ^2^ c. a. = coupling agent, a. p. = adhesion promoter, both added in given percentage per 10% SBP.

SBP		Talc	Additives		Tensile Modulus	Tensile Strength	Elongation at Break	Impact (Charpy)	
Type ^1^	%		c. a. ^2^	a. p. ^2^	MPa	MPa	%	Notched kJm^−2^	Unnotched kJm^−2^
PLA–PBS (4:3) composites									
-	0	16	0	0	3200 (2.4)	47.2 (1.2)	6.2 (24)	3.0 (14)	38.8 (11)
f	8	8	1	0	2960 (1.2)	44.4 (1.0)	6.4 (22)	3.1 (1.3)	24.0 (6.2)
f	8	8	1.5	0	2940 (1.5)	44.4 (0.8)	5.7 (14)	3.1 (2.7)	23.8 (8.7)
f	8	8	4	0	3020 (0.8)	44.3 (1.3)	4.0 (3.9)	2.4 (20)	21.8 (8.6)
f	8	8	6	0	3200 (1.1)	46.5 (1.1)	4.4(3.1)	2.4 (24)	20.9 (11)
f	16	0	1	0	2670 (1.5)	41.8 (1.3)	5.5 (25)	2.9 (2.4)	18.8 (15)
f	16	0	1	0.5	2750 (2.2)	41.6 (0.8)	4.1 (4.1)	2.0 (22)	16.0 (12)
f	16	0	1.5	0	2710 (1.2)	44.0 (0.9)	5.0 (13)	2.8 (10)	18.6 (7.8)
c	8	8	1	0	2950 (1.3)	43.7 (1.5)	4.2 (9.2)	3.1 (2.9)	13.4 (8.0)
c	8	8	1.5	0	3010 (1.2)	43.9 (1.0)	4.3 (7.3)	3.1 (1.8)	12.3 (15)
c	8	8	4	0	3600 (0.6)	41.6 (1.4)	3.2 (4.1)	2.4 (22)	13.2 (6.4)
c	16	0	1	0	2720 (1.6)	41.6 (0.5)	4.3 (6.7)	3.0 (2.5)	11.0 (11)
c	16	0	1	0.5	2750 (0.9)	38.1 (1.4)	3.4 (2.0)	2.4 (21)	10.4 (13)
PLA–PBS (3:4) composites									
-	0	16	0	0	2760 (1.2)	40.8 (1.7)	4.5 (8.5)	3.1 (2.8)	40.5 (20)
f	8	8	1	0	2470 (1.3)	39.3 (0.4)	5.8 (7.0)	2.5 (20)	27.4 (9.0)
f	16	0	1	0	2260 (0.7)	37.0 (0.6)	6.0 (12)	2.6 (16)	21.1 (14)
f	16	0	1	0.5	2730 (1.3)	41.5 (1.3)	4.0 (3.9)	1.7 (6.2)	16.1 (7.6)
f	16	0	4	0	2740 (1.4)	44.4 (0.9)	4.6 (3.0)	2.5 (19)	17.3 (11)
c	8	8	1	0	2480 (1.6)	38.1 (1.1)	4.5 (4.8)	2.9 (8.5)	16.0 (10)
c	16	0	1	0	2250 (0.2)	35.6 (1.5)	4.6 (6.1)	2.7 (12)	14.7 (18)
c	16	0	1	0.5	2680 (0.6)	37.3 (1.6)	3.4 (4.0)	2.4 (20)	10.4 (24)

**Table 6:** Calculated K-values according to Equation (4) of SBP composites with 25% SBP (PLA–PBS 9:1) or 16% SBP (PLA–PBS 4:3 or 3:4); ^1^ c. a. = coupling agent; a. p. = adhesion promoter, both added in given percentage per 10% SBP.

SBP	Additives		K	
%	c. a. ^1^	a. p. ^1^	Fine	Coarse
PLA–PBS (9:1)				
25	0.0	0.0	0.85	
25	0.5	0.0	0.67	0.77
25	1.0	0.0	0.51	0.59
25	1.5	0.0	0.38	0.52
25	0.0	0.5	0.51	
25	0.0	1.0	0.53	0.67
25	0.0	1.5	0.52	
25	1.0	0.5	0.37	0.70
25	1.0	1.0	0.39	0.59
25	1.0	1.5	0.22	0.58
25	2.0	0.5	0.28	
25	2.0	1.0	0.22	
25	2.0	1.5	0.24	
25	3.0	0.0	0.42	0.61
25	4.0	0.0	0.33	0.59
PLA–PBS (4:3)				
16	1.0	0.0	0.53	0.54
16	1.0	0.5	0.54	0.80
16	1.5	0.0	0.37	
PLA–PBS (3:4)				
16	1.0	0.0	0.66	0.77
16	1.0	0.5	0.30	0.63
16	4.0	0.0	0.07	

## Data Availability

Data are contained within the article.

## References

[1] Food and Agriculure Organisation of the United Nations FAOSTAT Data. http://fenix.fao.org/faostat/internal/en/#data/QCL.

[2] Rouilly A., Jorda J., Rigal L. (2006). Thermo-mechanical processing of sugar beet pulp. I. Twin-screw extrusion process. Carbohydr. Polym..

[3] Van der Poel P.W., Schiweck H., Schwartz T. (2000). Zuckertechnologie: Rüben- und Rohrzuckergewinnung.

[4] Van der Poel P.W., Schiweck H., Schwartz T. (1998). Sugar Technology: Beet and Cane Sugar Manufacture.

[5] Sun R., Hughes S. (1999). Fractional isolation and physico-chemical characterization of alkali-soluble polysaccharides from sugar beet pulp. Carbohydr. Polym..

[6] Knudsen K.E.B. (1997). Carbohydrate and lignin contents of plant masterials used in animal feeding. Anim. Feed. Sci. Technol..

[7] Brachi P., Riianova E., Miccio M., Miccio F., Ruoppolo G., Chirone R. (2017). Valorization of Sugar Beet Pulp via Torrefaction with a Focus on the Effect of the Preliminary Extraction of Pectins. Energy Fuels.

[8] Berłowska J., Pielech-Przybylska K., Balcerek M., Dziekońska-Kubczak U., Patelski P., Dziugan P., Kręgiel D. (2016). Simultaneous Saccharification and Fermentation of Sugar Beet Pulp for Efficient Bioethanol Production. BioMed Res. Int..

[9] Majzoobi M., Farahnaky A., Jamalian J., Mesbahi G., Sairi F., William P.A., Phillips G.O. (2010). Application of sugar beet pulp as dough and bread improver (Barbari bread). Gums and Stabilisers for the Food Industry 15, Proceedings of the 15th Gums and Stabilisers for the Food Industry Conference, Wrexham, UK, 22–26 June 2010.

[10] Michel F., Thibault J.-F., Barry J.-L., de Baynast R. (1988). Preparation and characterisation of dietary fibre from sugar beet pulp. J. Sci. Food Agric..

[11] Ačkar Đ., Jozinović A., Babić J., Miličević B., Panak Balentić J., Šubarić D. (2018). Resolving the problem of poor expansion in corn extrudates enriched with food industry by-products. Innov. Food Sci. Emerg. Technol..

[12] Dinand E., Chanzy H., Vignon M.R. (1996). Parenchymal cell cellulose from sugar beet pulp: Preparation and properties. Cellulose.

[13] Dinand E., Chanzy H., Vignon R.M. (1999). Suspensions of cellulose microfibrils from sugar beet pulp. Food Hydrocoll..

[14] Li M., Wang L., Li D., Cheng Y., Adhikari B. (2014). Preparation and characterization of cellulose nanofibers from de-pectinated sugar beet pulp. Carbohydr. Polym..

[15] Togrul H. (2003). Production of carboxymethyl cellulose from sugar beet pulp cellulose and rheological behaviour of carboxymethyl cellulose. Carbohydr. Polym..

[16] Oosterveld A. (2001). Isolation of feruloylated arabinans and rhamnogalacturonans from sugar beet pulp and their gel forming ability by oxidative cross-linking. Carbohydr. Polym..

[17] Huang X., Li D., Wang L. (2017). Characterization of pectin extracted from sugar beet pulp under different drying conditions. J. Food Eng..

[18] Turquois T., Rinaudo M., Taravel F., Heyraud A. (1999). Extraction of highly gelling pectic substances from sugar beet pulp and potato pulp: Influence of extrinsic parameters on their gelling properties. Food Hydrocoll..

[19] Cárdenas-Fernández M., Hamley-Bennett C., Leak D.J., Lye G.J. (2018). Continuous enzymatic hydrolysis of sugar beet pectin and l-arabinose recovery within an integrated biorefinery. Bioresour. Technol..

[20] Mathew S., Abraham T.E. (2004). Ferulic acid: An antioxidant found naturally in plant cell walls and feruloyl esterases involved in its release and their applications. Crit. Rev. Biotechnol..

[21] Micard V., Renard C., Thibault J.-F. (1994). Studies on Enzymic Release of Ferulic Acid from Sugar-Beet Pulp. LWT Food Sci. Technol..

[22] Kumar N., Pruthi V. (2014). Potential applications of ferulic acid from natural sources. Biotechnol. Rep..

[23] Rouilly A., Jorda J., Rigal L. (2006). Thermo-mechanical processing of sugar beet pulp. II. Thermal and rheological properties of thermoplastic SBP. Carbohydr. Polym..

[24] Rouilly A., Geneau-Sbartaï C., Rigal L. (2009). Thermo-mechanical processing of sugar beet pulp. III. Study of extruded films improvement with various plasticizers and cross-linkers. Bioresour. Technol..

[25] Liu B., Zhang J., Liu L., Hotchkiss A.T. (2011). Preparation and Properties of Water and Glycerol-plasticized Sugar Beet Pulp Plastics. J. Polym. Environ..

[26] Liu B., Zhang J., Liu L., Hotchkiss A.T. (2012). Utilization of Pectin Extracted Sugar Beet Pulp for Composite Application. J. Biobased Mater. Bioenergy.

[27] Liu L., Fishman M.L., Hicks K.B., Liu C.-K. (2005). Biodegradable composites from sugar beet pulp and poly(lactic acid). J. Agric. Food Chem..

[28] Liu L.S., Finkenstadt V.L., Liu C.-K., Coffin D.R., Willett J.L., Fishman M.L., Hicks K.B. (2007). Green Composites from Sugar Beet Pulp and Poly(lactic acid): Structural and Mechanical Characterization. J. Biobased Mat. Bioenergy.

[29] Finkenstadt V.L., Liu L., Willett J.L. (2007). Evaluation of Poly(lactic acid) and Sugar Beet Pulp Green Composites. J. Polym. Environ..

[30] Finkenstadt V.L., Liu C.-K., Cooke P.H., Liu L.S., Willett J.L. (2008). Mechanical Property Characterization of Plasticized Sugar Beet Pulp and Poly(Lactic Acid) Green Composites Using Acoustic Emission and Confocal Microscopy. J. Polym. Environ..

[31] Chen F., Liu L., Cooke P.H., Hicks K.B., Zhang J. (2008). Performance Enhancement of Poly(lactic acid) and Sugar Beet Pulp Composites by Improving Interfacial Adhesion and Penetration. Ind. Eng. Chem. Res..

[32] Mohamed A.A., Finkenstadt V.L., Palmquist D.E. (2008). Thermal properties of extruded/injection-molded poly(lactic acid) and biobased composites. J. Appl. Polym. Sci..

[33] Liu B., Bhaladhare S., Zhan P., Jiang L., Zhang J., Liu L., Hotchkiss A.T. (2011). Morphology and Properties of Thermoplastic Sugar Beet Pulp and Poly(butylene adipate-co-terepthalate) Blends. Ind. Eng. Chem. Res..

[34] Shen Z., Ghasemlou M., Kamdem D.P. (2015). Development and compatibility assessment of new composite film based on sugar beet pulp and polyvinyl alcohol intended for packaging applications. J. Appl. Polym. Sci..

[35] Tsuji H., Auras R., Lim L.-T., Selke S., Tsuji H. (2010). Hydrolytic Degradation. Poly(Lactic Acid): Synthesis, Structures, Properties, Processing, and Applications.

[36] Kopitzky R. Non-Published Results of an Internal FT-IR Study on Commercialized Coffee Capsules Mainly from Europe between 2019 and 2021.

[37] Su S., Kopitzky R., Tolga S., Kabasci S. (2019). Polylactide (PLA) and Its Blends with Poly(butylene succinate) (PBS): A Brief Review. Polymers.

[38] Aliotta L., Cinelli P., Coltelli M.B., Righetti M.C., Gazzano M., Lazzeri A. (2017). Effect of nucleating agents on crystallinity and properties of poly (lactic acid) (PLA). Eur. Polym. J..

[39] Lee H.L., Luner P. (1991). The Solubility Parameter of Cellulose and Alkyleneketene Dimer (AKD) Determined by Inverse Gas Chromatography. J. Wood Technol. Technol..

[40] Archer W.L. (1991). Determination of Hansen Solubility Parameters for selected Cellulose Ether Derivates. Ind. Eng. Chem. Res..

[41] Robeson L.M. (2007). Polymer Blends: A Comprehensive Review.

[42] Abbot S., Auras R., Lim L.-T., Selke S., Tsuji H. (2010). Chemical Compatibility of Poly(Lactic Acid): A Practical Framework Using Hansen Solubility Parameters. Poly(Lactic Acid): Synthesis, Structures, Properties, Processing, and Applications.

[43] Li H., Huneault M.A. (2007). Effect of nucleation and plasticization on the crystallization of poly(lactic acid). Polymer.

[44] Battegazore D., Bocchini S., Frache A. (2011). Crystallization kinetics of poly(lactic acid)-talc composites. Express Polym. Lett..

[45] Pivsa-Art W., Fujii K., Nomura K., Aso Y., Ohara H., Yamane H. (2016). Isothermal crystallization kinetics of talc-filled poly(lactic acid) and poly(butylene succinate) blends. J. Polym. Res..

[46] Agarval B.D., Broutman L.J., Chandrashekhara K. (2006). Analysis and Performance of Fibre.

[47] Yerbolat G., Amangeldi S., Ali H.M., Badanova N., Ashirbeok A., Islam G. Composite Materials Property Determination by Rule of Mixture and Monte Carlo Simulation. Proceedings of the 2018 IEEE International Conference on Advanced Manufacturing (ICAM).

[48] Tomar N., Maiti S.M. (2007). Mechanical Properties of PBT/ABAS Blends. J. Appl. Polym. Sci..

[49] Simões C.L., Viana J.C., Cunha A.M. (2009). Mechanical properties of poly(ε-caprolactone) and poly(lactic acid) blends. J. Appl. Polym. Sci..

[50] Cohen L.J., Ishai O. (1967). The Elastic Properties of Three Phase Composites. J. Compos. Mater..

[51] Halpin J.C., Kardos J.L. (1976). The Halpin-Tsai Equations: A Review. Polym. Eng. Sci..

[52] Nicolais L., Narkis M. (1971). Stress-Strain Behavior of Styrene-Acrylonitrile/Glass Bead Composites in the Glassy Region. Polym. Eng. Sci..

[53] Ramsteiner F., Theysohn R. (1984). On the tensile behaviour of filled composites. Composites.

